# Enhancing Large Language Model Reliability: Minimizing Hallucinations with Dual Retrieval-Augmented Generation Based on the Latest Diabetes Guidelines

**DOI:** 10.3390/jpm14121131

**Published:** 2024-11-30

**Authors:** Jaedong Lee, Hyosoung Cha, Yul Hwangbo, Wonjoong Cheon

**Affiliations:** 1 Healthcare AI Team, National Cancer Center, Goyang-si 10408, Gyeonggi-do, Republic of Koreakkido@ncc.re.kr (H.C.); yulhwangbo@ncc.re.kr (Y.H.); 2 Department of Cancer AI & Digital Health, Graduate School of Cancer Science and Policy, National Cancer Center, Goyang-si 10408, Gyeonggi-do, Republic of Korea; 3 Department of Radiation Oncology, Seoul St. Mary’s Hospital, College of Medicine, The Catholic University of Korea, Seoul 06591, Republic of Korea

**Keywords:** large language models, retrieval-augmented generation, diabetes management, medical information retrieval, ensemble retriever

## Abstract

**Background/Objectives:** Large language models (LLMs) show promise in healthcare but face challenges with hallucinations, particularly in rapidly evolving fields like diabetes management. Traditional LLM updating methods are resource-intensive, necessitating new approaches for delivering reliable, current medical information. This study aimed to develop and evaluate a novel retrieval system to enhance LLM reliability in diabetes management across different languages and guidelines. **Methods:** We developed a dual retrieval-augmented generation (RAG) system integrating both Korean Diabetes Association and American Diabetes Association 2023 guidelines. The system employed dense retrieval with 11 embedding models (including OpenAI, Upstage, and multilingual models) and sparse retrieval using BM25 algorithm with language-specific tokenizers. Performance was evaluated across different top-k values, leading to optimized ensemble retrievers for each guideline. **Results:** For dense retrievers, Upstage’s Solar Embedding-1-large and OpenAI’s text-embedding-3-large showed superior performance for Korean and English guidelines, respectively. Multilingual models outperformed language-specific models in both cases. For sparse retrievers, the ko_kiwi tokenizer demonstrated superior performance for Korean text, while both ko_kiwi and porter_stemmer showed comparable effectiveness for English text. The ensemble retrievers, combining optimal dense and sparse configurations, demonstrated enhanced coverage while maintaining precision. **Conclusions:** This study presents an effective dual RAG system that enhances LLM reliability in diabetes management across different languages. The successful implementation with both Korean and American guidelines demonstrates the system’s cross-regional capability, laying a foundation for more trustworthy AI-assisted healthcare applications.

## 1. Introduction

Large language models (LLMs) have revolutionized natural language processing and artificial intelligence, demonstrating remarkable capabilities in understanding and generating human-like text across various domains. Both open-source models, like T5 (Google, Mountain View, CA, USA) [1] and LLAMA (Meta, Menlo Park, CA, USA) [2], and commercial models, such as ChatGPT (OpenAI, San Francisco, CA, USA) [3] and Claude (Anthropic, San Francisco, CA, USA) [4], have shown promise in multiple applications, from creative writing to complex question answering. However, the challenge of hallucination—the generation of factually incorrect content—remains a significant concern, particularly in fields requiring high accuracy and reliability like healthcare [5].

In medical contexts, hallucination refers to the generation of inaccurate medical information that could lead to improper clinical decision supports to physician. For example, an LLM might incorrectly state medication dosages or combine unrelated treatment guidelines, potentially compromising patient care.

The medical domain’s expansive and dynamic corpus of knowledge presents a challenge for LLMs to maintain contemporaneity. Several factors contribute to this challenge, including training on potentially inaccurate or outdated information, the statistical nature of language model prediction, and the lack of access to external, up-to-date knowledge bases during inference [6,7,8,9]. While various approaches like full-parameter fine-tuning, parameter-efficient methods (e.g., Prefix-tunning, LoRA, Adapter), and knowledge distillation have been explored [10,11,12,13]. These methods require additional training and computational resources, making them resource-intensive and costly [14,15]. This constraint underscores the necessity for alternative methodologies, such as retrieval-augmented generation (RAG) systems, to sustain current and reliable AI-assisted medical information without necessitating frequent model recalibration. 

Diabetes management presents an ideal case study for evaluating LLM reliability due to its complex, multifaceted nature. Guidelines must address various aspects, including screening criteria, risk assessment, treatment selection, and monitoring protocols, all of which require precise interpretation and application. The frequent updates to these guidelines, driven by new clinical evidence and therapeutic advances, make it particularly challenging for LLMs to maintain accuracy without external knowledge sources. 

Our analysis encompasses both the Korean Diabetes Association’s 2023 Clinical Practice Guidelines [16] and the American Diabetes Association’s Standards of Care in Diabetes—2023 [17], representing diverse regional perspectives and approaches. The Korean guidelines introduce significant changes such as lowering the recommended age for diabetes screening from 40 to 35 years and expanding criteria for identifying high-risk individuals. Similarly, the American guidelines emphasize novel therapeutic approaches, particularly in cardiovascular risk management and the use of GLP-1 receptor agonists. Both guidelines stress personalized care approaches and continuous glucose monitoring, highlighting the global convergence in diabetes care standards while maintaining region-specific considerations.

While single RAG systems employ a retrieval step using either dense or sparse methods, each approach has inherent limitations. Dense retrievers excel at capturing semantic relationships but may miss exact keyword matches crucial for medical terminology, while sparse retrievers effectively find specific terms but often miss conceptually related information. For example, when querying about diabetes treatments, a dense retriever might understand the concept of “glucose management” but miss specific medication names, while a sparse retriever might find exact medication names but miss related contraindications [18,19,20,21].

Our proposed dual RAG system addresses these limitations by combining dense semantic search for conceptual understanding with sparse keyword-based retrieval for medical guideline details. This approach aims to provide more comprehensive and reliable responses in complex medical domains, improving the accuracy of AI-assisted healthcare information.

## 2. Materials and Methods

### 2.1. Corpus Preprocessing

We utilized two primary data sources: the 2023 Korean Diabetes Association (KDA) guidelines and the American Diabetes Association (ADA)’s Standards of Care in Diabetes—2023. The preprocessing was facilitated by AutoRAG (Markr.AI, Seoul, Republic of Korea) [22], an AutoML tool designed for automatically finding and optimizing RAG pipelines. This tool was chosen for its efficiency in evaluating various RAG modules and optimizing the pipeline for our data and use-case.

For each guideline document, we implemented independent preprocessing pipelines to maintain regional context integrity. To break down the guidelines into manageable segments, we set the chunk size to 1000 characters, with an overlap of 200 characters (1/5 of chunk size) between adjacent chunks. This chunk size was chosen to balance between maintaining sufficient context for complex medical concepts while keeping segments focused enough for precise retrieval. The overlap helps preserve context at chunk boundaries, ensuring that related information is not artificially separated.

The resulting corpus was structured into a tabular format with four primary columns (Table 1): index (a sequential identifier for each chunk), doc_id (a unique identifier for each document segment), contents (the actual text of the chunk), and metadata (additional information about the chunk, including creation date and source file details).

This structured approach to corpus preprocessing allows for efficient retrieval and context preservation. It forms the foundation for subsequent stages in the AutoRAG pipeline, including the evaluation of generating question-answer (QA) pairs and the evaluation of various retriever models.

### 2.2. Question–Answer Pair Creation

To create a comprehensive QA dataset based on the preprocessed corpus, we employed ChatGPT-4.o (OpenAI, San Francisco, CA, USA), leveraging its advanced language understanding and generation capabilities. We implemented a few-shot learning technique to enhance the quality and relevance of the generated QA pairs. This approach involved providing ChatGPT-4.o with carefully crafted example QA pairs, which guided the model in generating contextually appropriate and clinically relevant QA pairs. This method allowed us to create a dataset that accurately reflected the content and complexity of the diabetes management guidelines while ensuring a wide coverage of relevant topics.

The generated QA pairs underwent rigorous validation by a panel of medical experts specializing in diabetes care. Each QA pair was evaluated for clinical accuracy, relevance to current guidelines, and practical utility in patient care. Only pairs that received unanimous approval from the expert panel were included in the final dataset. This validation process ensured that the QA pairs accurately reflected both the Korean and American diabetes guidelines while maintaining clinical relevance.

The resulting QA dataset was structured with four main columns: qid (a unique identifier for each QA pair), retrieval_gt (ground truth document ID for retrieval), query (the question), and generation_gt (the answer). Table 2 provides an example of the QA dataset format.

### 2.3. Retriever Evaluation

The retriever evaluation encompassed both sparse and dense retrieval methods. For dense retrieval, we evaluated multiple models including OpenAI embedding models (text-embedding-ada-002, text-embedding-3-small, text-embedding-3-large), the Upstage embedding model (Solar Embedding-1-large), Korean-specific models (ko-sroberta-multitask, KoSimCSE-roberta), multilingual models (paraphrase-multilingual-mpnet-base-v2, paraphrase-multilingual-MiniLM-L12-v2, multilingual-e5-large-instruct), and task-specific models (kf-deberta-multitask, gte-multilingual-base).

For sparse retrieval, we implemented the BM25 algorithm with two distinct tokenizers: ko_kiwi tokenizer specifically designed for Korean text processing, and porter_stemmer tokenizer commonly used for text stemming. We applied both tokenizers to both KDA and ADA guidelines to evaluate their retrieval performance across different language contexts. The ko_kiwi tokenizer specializes in Korean morphological analysis and word segmentation, while the porter_stemmer reduces words to their root form. This comparative approach aimed to assess the effectiveness of different tokenization strategies in retrieving relevant information from guidelines in different languages.

Our evaluation methodology employed a top-k retrieval approach, with k values set at 1, 3, 5, 10, and 50. To ensure a thorough assessment of retrieval performance, we utilized a comprehensive set of evaluation metrics including retrieval f1-score, recall, precision, mean average precision (MAP), mean reciprocal rank (MRR), and normalized discounted cumulative gain (NDCG).

These metrics were chosen for their ability to capture different aspects of retrieval performance, particularly in medical information retrieval where both precision and recall are crucial. Combining diverse retriever models, varied k values, and comprehensive metrics allowed us to evaluate retrieval performance thoroughly.

## 3. Results

### 3.1. Dense Retriever

Our evaluation of various dense retriever models revealed significant performance differences across multiple metrics. We tested a range of embedding models, including language-specific (e.g., KoSimCSE-roberta, ko-sroberta-multitask), multilingual (e.g., gte-multilingual-base, multilingual-e5-large-instruct), and general-purpose models (e.g., OpenAI’s text-embedding models, Upstage’s Solar Embedding-1-large) on both KDA and ADA guidelines. The performance details are tabulated in Table 3 for KDA and Table 4 for ADA results.

**Figure 1:**
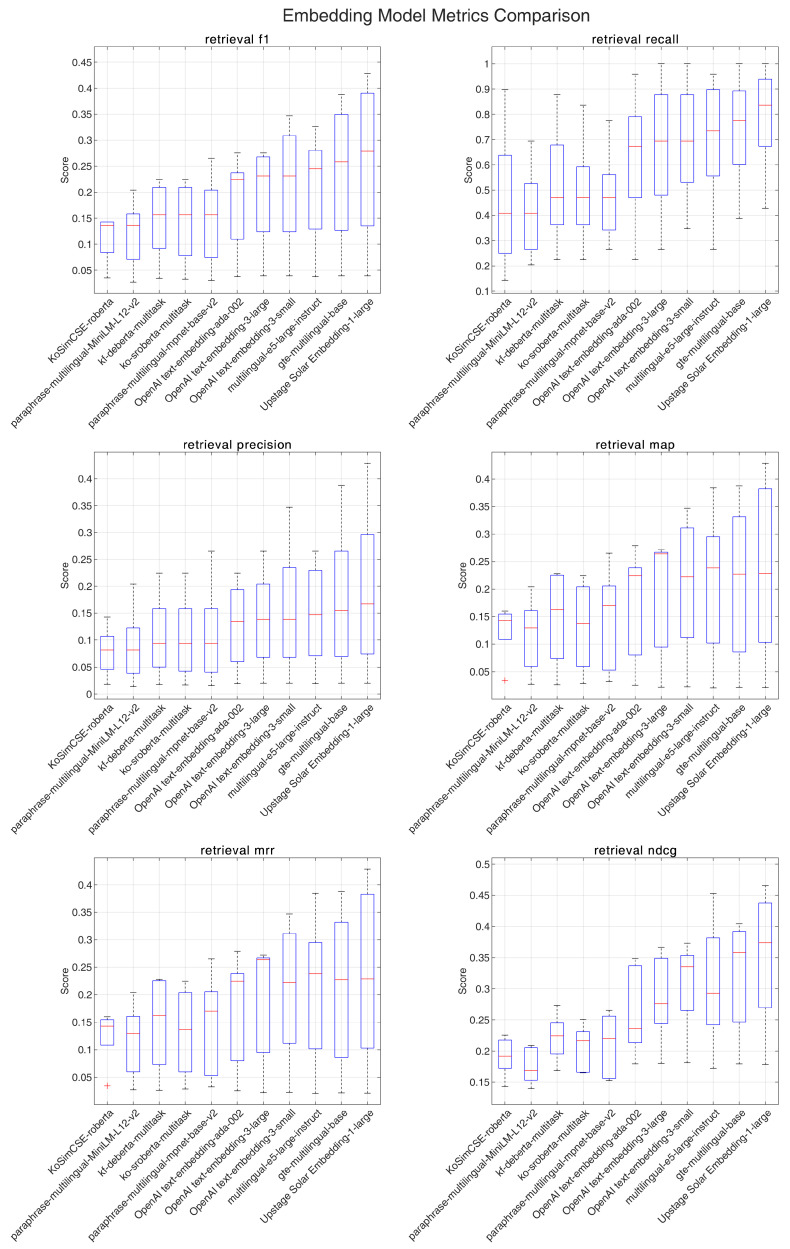
Performance comparison of various embedding models across multiple retrieval metrics for the Korean Diabetes Association’s guideline.

**Figure 2:**
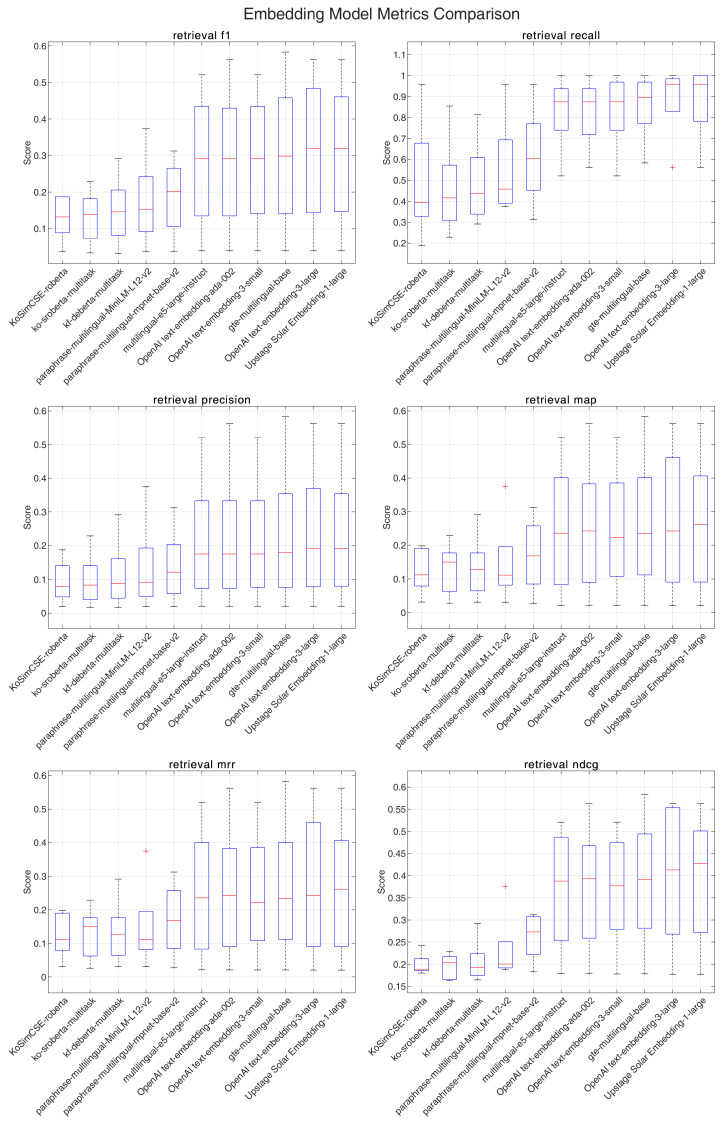
Performance comparison of various embedding models across multiple retrieval metrics for the American Diabetes Association’s Standards of Care in Diabetes.

Figure 1 and Figure 2 comprehensively compare the performance metrics for each embedding model, illustrating the distribution of scores for retrieval f1-score, recall, precision, MAP, MRR, and NDCG.

When averaging across all k values, distinct performance patterns emerged for each guideline. For the KDA guidelines, the Upstage embedding model (Solar Embedding-1-large) demonstrated the strongest performance with an f1-score of 0.258, recall of 0.788, precision of 0.192, MAP and MRR both at 0.235, and NDCG of 0.349. In contrast, for the ADA guidelines, which generally showed higher metric scores overall, OpenAI’s text-embedding-3-large achieved superior performance with an f1-score of 0.312, recall of 0.883, precision of 0.236, MAP and MRR both at 0.273, and NDCG of 0.400.

Cross-guideline analysis revealed several interesting patterns. General-purpose models showed robust performance across both languages, with Upstage embed and OpenAI models consistently performing well. Multilingual models, particularly gte-multilingual-base, demonstrated consistent performance across both Korean and English texts. Notably, language-specific models showed relatively lower performance even in Korean text, suggesting that broader language understanding capabilities may be more beneficial for medical domain retrieval tasks. Overall performance metrics were consistently higher for English text retrieval compared to Korean text retrieval.

The analysis of k-value effects revealed distinct patterns between the guidelines. For ADA guidelines, at k = 10, multiple models achieved recall rates exceeding 0.95, while KDA guidelines showed more moderate recall rates, with the highest being around 0.92. In both cases, increasing k values led to higher recall at the cost of precision, resulting in lower f1-scores.

Execution time analysis showed that language-specific and multilingual models generally demonstrated faster processing speeds compared to general-purpose models across both guidelines. The Upstage embed and OpenAI models typically required longer processing times, though this trade-off was balanced by their superior retrieval performance. These execution time patterns remained consistent across different k values, with variations in processing time generally correlating with model architecture complexity rather than guideline language or k-value selection.

### 3.2. Sparse Retriever

For our sparse retrieval evaluation, we implemented the BM25 algorithm using two different tokenizers: ko_kiwi tokenizer specifically designed for Korean text, and porter_stemmer for English text. This comparative approach allowed us to evaluate the effectiveness of tokenization strategies across different languages in our diabetes management guidelines.

We evaluated the BM25 algorithm’s performance across different top-k values (1, 3, 5, 10, and 50) for both KDA and ADA guidelines. The results are summarized in Table 5 for KDA and Table 6 for ADA.

Our analysis revealed distinct patterns between the two guidelines. For KDA guidelines, the ko_kiwi tokenizer demonstrated superior performance compared to the porter_stemmer across all metrics. Starting with a higher f1-score of 0.306 compared to porter_stemmer’s 0.245 at k = 1, it consistently maintained better performance as k increased. At k = 50, it achieved a high recall of 0.98 with better MAP (0.425) and NDCG (0.542) scores compared to porter_stemmer’s respective scores (0.386 and 0.497), indicating more effective retrieval of relevant documents for Korean text.

For ADA guidelines, the ko_kiwi tokenizer showed strong performance across metrics, achieving an f1-score of 0.542 at k = 1, and maintaining competitive performance as k increased. At k = 10, it achieved a recall of 0.896 while still maintaining reasonable precision. The porter_stemmer showed comparable performance, particularly in recall metrics at higher k values, reaching 1.000 at k = 50.

Across both guidelines, we observed consistent trends: as the top-k value increased, recall, MAP, MRR, and NDCG generally improved, while precision decreased. This trade-off was reflected in the f1-scores, which typically decreased with higher k values despite improved recall.

A notable difference emerged in execution times between tokenizers. The ko_kiwi tokenizer consistently required around 1.0–1.1 s of processing time across different k values, while the porter_stemmer was significantly faster, typically requiring only 0.001–0.006 s. This performance difference suggests a trade-off between processing speed and tokenization sophistication, particularly relevant for Korean text processing.

### 3.3. Ensemble Retriever

Our evaluation revealed distinct performance characteristics between dense and sparse retrievers across both the KDA and ADA guidelines. For KDA guidelines, the dense retriever using the Upstage embedding model (Solar Embedding-1-large) demonstrated superior performance across all metrics at top-k = 3. For ADA guidelines, OpenAI’s text-embedding-3-large achieved optimal performance at top-k = 1, with consistent scores of 0.563 across all metrics (f1-score, recall, precision, MAP, MRR, and NDCG). The sparse retriever (BM25) exhibited varying characteristics depending on the tokenizer and guideline language: for Korean text, the ko_kiwi tokenizer demonstrated superior performance, while for English text, both ko_kiwi and porter_stemmer tokenizers showed comparable results.

Based on these findings, we developed separate ensemble retrievers optimized for each guideline. For KDA guidelines, we combined the high-precision dense retriever (Solar Embedding-1-large, top-k = 3) with the sparse retriever (BM25 with ko_kiwi tokenizer, top-k = 50). For ADA guidelines, we integrated OpenAI’s text-embedding-3-large (top-k = 1) with the BM25 retriever (top-k = 10 with porter_stemmer tokenizer).

The ensemble method combines retrieved documents from both approaches, merging their unique contributions to create a more comprehensive result set. This straightforward combination strategy leverages the complementary strengths of both retrieval methods: the precision of dense retrievers and the broad coverage of sparse retrievers.

The ensemble approach demonstrates significant potential for improved overall performance, particularly in recall and ranking quality, while maintaining precision. Although it introduces some computational overhead, primarily from the ko_kiwi tokenizer processing time in the sparse retriever component, the performance gains justify this trade-off, especially in critical applications like medical information retrieval where both accuracy and comprehensiveness are essential.

## 4. Discussion

This study aimed to enhance the reliability of LLM and minimize hallucinations in diabetes management through the development of a dual RAG system. Our research addresses the critical need for accurate and up-to-date information retrieval in medical applications. The key contributions include the development of a dual RAG directly combining dense (semantic) and sparse (keyword-based) retrieval, integration of current medical guidelines, implementation of robust safeguards to minimize hallucinations, and a comprehensive evaluation framework.

Our findings revealed significant performance differences between dense and sparse retrieval methods across different language guidelines. For Korean text (KDA guidelines), the dense retriever using the Upstage embedding model (Solar Embedding-1-large) demonstrated optimal performance at top-k = 3, while for English text (ADA guidelines), OpenAI’s text-embedding-3-large achieved the best results at top-k = 1. Notably, multilingual and general-purpose models outperformed language-specific models in both guidelines, suggesting that broader language understanding capabilities may be more advantageous for specialized medical domains.

The sparse retriever exhibited distinct characteristics based on the tokenizer choice and guideline language. The ko_kiwi tokenizer demonstrated superior performance for Korean text, while both ko_kiwi and porter_stemmer performed comparably for English text. 

The trade-off between recall and precision with varying top-k values is particularly significant in medical contexts where missing critical information (false negatives) could have more serious consequences than including some irrelevant information (false positives). For instance, when retrieving information about medication contraindications, it is preferable to retrieve all potentially relevant warnings, even if some are not directly applicable.

However, our study has limitations. While RAG systems offer advantages over parameter-efficient fine-tuning methods in maintaining contemporaneous information without model retraining, they present different computational trade-offs. Unlike parameter-efficient fine-tuning approaches that require significant computational resources during the training phase, RAG systems incur increased computational costs and memory requirements during each inference, particularly evident in the processing time differences between tokenizers in our sparse retrieval implementation. Additionally, the fixed GPU memory constraints create a trade-off between retrieved context length and input length—as the size of retrieved documents increases through RAG, the available space for user input necessarily decreases.

A significant challenge we encountered involves the OCR processing of medical documents with varying structures and the accurate conversion of tables and figures into textual format. This transformation process is crucial for maintaining the integrity and accessibility of medical information but remains technically challenging.

Our research demonstrates potential applicability beyond diabetes management. The successful implementation with both KDA and ADA guidelines validates the system’s cross-regional capability and suggests broader applicability. This approach could be extended to other medical fields with regularly updated guidelines, such as oncology treatment protocols or cardiology practice standards. The system’s demonstrated ability to effectively handle guidelines from different regions while maintaining accuracy indicates its potential for broader geographical implementation.

For successful integration into clinical workflows, implementing dual RAG systems with on-premise LLMs emerges as a preferred approach. This strategy ensures data privacy, reduces latency, and maintains compliance with healthcare regulations while providing the benefits of our dual retrieval system.

Future research directions should explore advanced ensemble techniques for enhanced retrieval performance, while expanding system capabilities through a multi-layered LLM architecture. The introduction of interpreter LLMs could facilitate seamless access to international guidelines across languages, while specialized LLMs managing distinct guideline domains would maintain expertise in their respective areas. A coordinating agent LLM could then synthesize comprehensive medical solutions by integrating insights across these specialized components. This architectural approach would preserve both contemporaneity and accuracy across diverse medical topics while enabling broader geographical reach. Comprehensive evaluation across expanded medical domains and diverse clinical datasets would validate the system’s scalability and real-world applicability.

In conclusion, this study demonstrates the potential of advanced retrieval methods, particularly our ensemble approach, to enhance the reliability of LLMs in specialized medical domains. By minimizing hallucinations and improving the accuracy of information retrieval, we contribute to ongoing efforts to make AI-assisted healthcare more trustworthy and effective. Our work paves the way for future advancements in applying LLMs to complex and critical fields such as medicine, where accuracy and reliability are paramount.

## 5. Conclusions

This study presents a dual RAG system that enhances the accuracy of LLMs in diabetes management by combining dense retrievers (Solar Embedding-1-large, OpenAI’s text-embedding-3-large) with sparse BM25 retrieval. Our evaluation across Korean and American diabetes guidelines demonstrated that this ensemble approach effectively reduces hallucinations while maintaining high retrieval performance. The system’s successful implementation across different languages and guidelines, particularly its strong performance with general-purpose models in specialized medical domains, demonstrates its potential for broader healthcare applications. This work lays a foundation for more trustworthy AI-assisted healthcare, offering a practical approach to enhancing clinical decision support while maintaining the contemporaneity of medical knowledge without frequent model retraining.

## Figures and Tables

**Table 1:** Three examples of corpus extracted from the Korean Diabetes Association Guidelines.

Index	Doc_Id	Contents	Metadata
9	DOC_UID#9	According to the Diabetes Prevention Study, among 446 subjects newly diagnosed with diabetes and with a body mass index of 23 kg/m^2^ or higher, 76.2% had fasting plasma glucose levels below 126 mg/dL, and 59.2% exceeded only the 2 h plasma glucose criteria after glucose loading. Fasting plasma glucose and HbA1c were below the criteria. When fasting plasma glucose was below 100 mg/dL (normal), …	‘creation_date’: ‘YYYY-MM-DD’, ‘file_name’: ‘chapter_name.docx’, ‘file_path’: ‘/path/to/file’
25	DOC_UID#25	effectively reduces the failure of glycemic control and achieves. It was significantly higher than monotherapy. The occurrence of hypoglycemia was also very mild. The results of the VERIFY study can be interpreted as showing that early combination therapy effectively reduces the failure of glycemic control and achieves glycemic control targets for a longer period. However, it does not provide evidence on whether glycemic control lower than HbA1c 6.5% is necessary. Also, whether early combination therapy can reduce the incidence of complications…	‘creation_date’: ‘YYYY-MM-DD’, ‘file_name’: ‘chapter_name.docx’, ‘file_path’: ‘/path/to/file’
306	DOC_UID#306	Generated Questions Index: 0 Answer Diabetes is named for the excretion of glucose (glycosuria) in urine. In normal individuals, blood glucose is regulated within a narrow range so that glucose does not overflow into the urine. The hormone ‘insulin’ secreted by the pancreas plays an important role. When this insulin is deficient or does not work properly…	‘creation_date’: ‘YYYY-MM-DD’, ‘file_name’: ‘chapter_name.docx’, ‘file_path’: ‘/path/to/file’

Korean translated into English.

**Table 2:** Three examples of question–answer data generated from the extracted corpus data.

Qid	Retrieval_Gt	Query	Generation_Gt
UID#1	[array([ ‘DOC_UID#9], dtype = object)]	What tests should women diagnosed with gestational diabetes undergo after childbirth?	Women diagnosed with gestational diabetes should have a 75 g oral glucose tolerance test 4–12 weeks after delivery to check their glucose tolerance status. If the test results are normal, annual diabetes screening should be considered.
UID#2	[array([ ‘DOC_UID#25’], dtype = object)]	Why should the HbA1c target be maintained below 6.5%?	HbA1c reflects average blood glucose levels over the past 2–3 months. Maintaining HbA1c below 6.5% can reduce the risk of complications. However, the target may be adjusted based on individual conditions.
UID#3	[array( [‘DOC_UID#306’], dtype = object)]	Why does urine volume increase in diabetes?	In diabetes, glucose is excreted in urine. As glucose draws water with it, urine volume increases. This results in dehydration, which causes severe thirst.

**Table 3:** Results of quantitative analysis for dense retrievers: Korean Diabetes Association guideline.

Embedding_Model	Top_k	Execution Time (s)	f1-Score	Recall	Precision	MAP	MRR	NDCG
ko-sroberta-multitask	1	0.063	0.224	0.224	0.224	0.224	0.224	0.224
openai_embed_3_large	1	0.033	0.265	0.265	0.265	0.265	0.265	0.265
gte-multilingual-base	1	0.093	0.388	0.388	0.388	0.388	0.388	0.388
kf-deberta-multitask	1	0.044	0.224	0.224	0.224	0.224	0.224	0.224
paraphrase-multilingual-mpnet-base-v2	1	0.061	0.265	0.265	0.265	0.265	0.265	0.265
openai_embed_3_small	1	0.024	0.347	0.347	0.347	0.347	0.347	0.347
paraphrase-multilingual-MiniLM-L12-v2	1	0.055	0.204	0.204	0.204	0.204	0.204	0.204
multilingual-e5-large-instruct	1	0.088	0.265	0.265	0.265	0.265	0.265	0.265
KoSimCSE-roberta	1	0.022	0.143	0.143	0.143	0.143	0.143	0.143
openai	1	0.022	0.224	0.224	0.224	0.224	0.224	0.224
upstage_embed	1	0.078	0.429	0.429	0.429	0.429	0.429	0.429
ko-sroberta-multitask	3	0.003	0.204	0.408	0.136	0.197	0.197	0.251
openai_embed_3_large	3	0.028	0.276	0.551	0.184	0.272	0.272	0.343
gte-multilingual-base	3	0.003	0.337	0.673	0.224	0.313	0.313	0.404
kf-deberta-multitask	3	0.003	0.204	0.408	0.136	0.228	0.228	0.273
paraphrase-multilingual-mpnet-base-v2	3	0.003	0.184	0.367	0.122	0.170	0.170	0.220
openai_embed_3_small	3	0.022	0.296	0.592	0.197	0.299	0.299	0.373
paraphrase-multilingual-MiniLM-L12-v2	3	0.002	0.143	0.286	0.095	0.129	0.129	0.169
multilingual-e5-large-instruct	3	0.004	0.327	0.653	0.218	0.384	0.384	0.453
KoSimCSE-roberta	3	0.003	0.143	0.286	0.095	0.160	0.160	0.192
openai	3	0.021	0.276	0.551	0.184	0.279	0.279	0.348
upstage_embed	3	0.063	0.378	0.755	0.252	0.367	0.367	0.465
ko-sroberta-multitask	5	0.003	0.156	0.469	0.094	0.137	0.137	0.216
openai_embed_3_large	5	0.026	0.231	0.694	0.139	0.264	0.264	0.367
gte-multilingual-base	5	0.003	0.259	0.776	0.155	0.227	0.227	0.358
kf-deberta-multitask	5	0.004	0.156	0.469	0.094	0.162	0.162	0.236
paraphrase-multilingual-mpnet-base-v2	5	0.003	0.156	0.469	0.094	0.186	0.186	0.253
openai_embed_3_small	5	0.023	0.231	0.694	0.139	0.222	0.222	0.335
paraphrase-multilingual-MiniLM-L12-v2	5	0.003	0.136	0.408	0.082	0.146	0.146	0.209
multilingual-e5-large-instruct	5	0.004	0.245	0.735	0.147	0.238	0.238	0.358
KoSimCSE-roberta	5	0.003	0.136	0.408	0.082	0.153	0.153	0.215
openai	5	0.016	0.224	0.673	0.135	0.225	0.225	0.333
upstage_embed	5	0.059	0.279	0.837	0.167	0.229	0.229	0.374
ko-sroberta-multitask	10	0.004	0.093	0.510	0.051	0.070	0.070	0.165
openai_embed_3_large	10	0.028	0.152	0.837	0.084	0.119	0.119	0.276
gte-multilingual-base	10	0.004	0.156	0.857	0.086	0.107	0.107	0.269
kf-deberta-multitask	10	0.004	0.111	0.612	0.061	0.089	0.089	0.204
paraphrase-multilingual-mpnet-base-v2	10	0.004	0.089	0.490	0.049	0.060	0.060	0.153
openai_embed_3_small	10	0.024	0.152	0.837	0.084	0.142	0.142	0.293
paraphrase-multilingual-MiniLM-L12-v2	10	0.003	0.085	0.469	0.047	0.071	0.071	0.158
multilingual-e5-large-instruct	10	0.005	0.160	0.878	0.088	0.129	0.129	0.293
KoSimCSE-roberta	10	0.004	0.100	0.551	0.055	0.133	0.133	0.226
openai	10	0.023	0.134	0.735	0.073	0.098	0.098	0.236
upstage_embed	10	0.046	0.167	0.918	0.092	0.130	0.130	0.300
ko-sroberta-multitask	50	0.009	0.033	0.837	0.017	0.029	0.029	0.166
openai_embed_3_large	50	0.034	0.039	1.000	0.020	0.022	0.022	0.180
gte-multilingual-base	50	0.008	0.039	1.000	0.020	0.022	0.022	0.179
kf-deberta-multitask	50	0.009	0.034	0.878	0.018	0.026	0.026	0.169
paraphrase-multilingual-mpnet-base-v2	50	0.008	0.030	0.776	0.016	0.032	0.032	0.157
openai_embed_3_small	50	0.028	0.039	1.000	0.020	0.022	0.022	0.181
paraphrase-multilingual-MiniLM-L12-v2	50	0.008	0.027	0.694	0.014	0.027	0.027	0.140
multilingual-e5-large-instruct	50	0.009	0.038	0.959	0.019	0.021	0.021	0.172
KoSimCSE-roberta	50	0.008	0.035	0.898	0.018	0.034	0.034	0.182
openai	50	0.027	0.038	0.959	0.019	0.025	0.025	0.179
upstage_embed	50	0.054	0.039	1.000	0.020	0.021	0.021	0.179

Mean average precision (MAP); mean reciprocal rank (MRR); and normalized discounted cumulative gain (NDCG).

**Table 4:** Results of quantitative analysis for dense retrievers: American Diabetes Association guideline.

Embedding_Model	Top_k	Execution Time (s)	f1-Score	Recall	Precision	MAP	MRR	NDCG
ko-sroberta-multitask	1	0.003	0.229	0.229	0.229	0.229	0.229	0.229
openai_embed_3_large	1	0.027	0.583	0.583	0.583	0.583	0.583	0.583
gte-multilingual-base	1	0.003	0.563	0.563	0.563	0.563	0.563	0.563
kf-deberta-multitask	1	0.004	0.292	0.292	0.292	0.292	0.292	0.292
paraphrase-multilingual-mpnet-base-v2	1	0.003	0.313	0.313	0.313	0.313	0.313	0.313
openai_embed_3_small	1	0.032	0.521	0.521	0.521	0.521	0.521	0.521
paraphrase-multilingual-MiniLM-L12-v2	1	0.002	0.375	0.375	0.375	0.375	0.375	0.375
multilingual-e5-large-instruct	1	0.005	0.521	0.521	0.521	0.521	0.521	0.521
KoSimCSE-roberta	1	0.003	0.188	0.188	0.188	0.188	0.188	0.188
openai	1	0.048	0.563	0.563	0.563	0.563	0.563	0.563
upstage_embed	1	0.074	0.563	0.563	0.563	0.563	0.563	0.563
ko-sroberta-multitask	3	0.003	0.167	0.333	0.111	0.160	0.160	0.203
openai_embed_3_large	3	0.038	0.458	0.917	0.306	0.427	0.427	0.551
gte-multilingual-base	3	0.003	0.417	0.833	0.278	0.340	0.340	0.465
kf-deberta-multitask	3	0.004	0.177	0.354	0.118	0.139	0.139	0.193
paraphrase-multilingual-mpnet-base-v2	3	0.003	0.250	0.500	0.167	0.240	0.240	0.305
openai_embed_3_small	3	0.029	0.406	0.813	0.271	0.340	0.340	0.460
paraphrase-multilingual-MiniLM-L12-v2	3	0.003	0.198	0.396	0.132	0.135	0.135	0.201
multilingual-e5-large-instruct	3	0.005	0.406	0.813	0.271	0.361	0.361	0.475
KoSimCSE-roberta	3	0.003	0.188	0.375	0.125	0.198	0.198	0.243
openai	3	0.028	0.385	0.771	0.257	0.323	0.323	0.436
upstage_embed	3	0.081	0.427	0.854	0.285	0.354	0.354	0.481
ko-sroberta-multitask	5	0.003	0.139	0.417	0.083	0.150	0.150	0.213
openai_embed_3_large	5	0.026	0.319	0.958	0.192	0.243	0.243	0.413
gte-multilingual-base	5	0.003	0.299	0.896	0.179	0.235	0.235	0.391
kf-deberta-multitask	5	0.004	0.146	0.438	0.088	0.128	0.128	0.201
paraphrase-multilingual-mpnet-base-v2	5	0.003	0.201	0.604	0.121	0.168	0.168	0.273
openai_embed_3_small	5	0.028	0.292	0.875	0.175	0.222	0.222	0.377
paraphrase-multilingual-MiniLM-L12-v2	5	0.003	0.153	0.458	0.092	0.111	0.111	0.193
multilingual-e5-large-instruct	5	0.005	0.292	0.875	0.175	0.236	0.236	0.388
KoSimCSE-roberta	5	0.004	0.132	0.396	0.079	0.112	0.112	0.180
openai	5	0.034	0.292	0.875	0.175	0.243	0.243	0.393
upstage_embed	5	0.074	0.319	0.958	0.192	0.262	0.262	0.427
ko-sroberta-multitask	10	0.004	0.087	0.479	0.048	0.075	0.075	0.163
openai_embed_3_large	10	0.025	0.178	0.979	0.098	0.114	0.114	0.299
gte-multilingual-base	10	0.004	0.174	0.958	0.096	0.142	0.142	0.316
kf-deberta-multitask	10	0.004	0.098	0.542	0.054	0.076	0.076	0.178
paraphrase-multilingual-mpnet-base-v2	10	0.004	0.129	0.708	0.071	0.104	0.104	0.236
openai_embed_3_small	10	0.025	0.174	0.958	0.096	0.137	0.137	0.313
paraphrase-multilingual-MiniLM-L12-v2	10	0.003	0.110	0.604	0.060	0.099	0.099	0.209
multilingual-e5-large-instruct	10	0.005	0.167	0.917	0.092	0.104	0.104	0.277
KoSimCSE-roberta	10	0.004	0.106	0.583	0.058	0.095	0.095	0.203
openai	10	0.044	0.167	0.917	0.092	0.114	0.114	0.285
upstage_embed	10	0.073	0.182	1.000	0.100	0.114	0.114	0.304
ko-sroberta-multitask	50	0.008	0.033	0.854	0.017	0.027	0.027	0.166
openai_embed_3_large	50	0.033	0.039	1.000	0.020	0.020	0.020	0.177
gte-multilingual-base	50	0.007	0.039	1.000	0.020	0.021	0.021	0.178
kf-deberta-multitask	50	0.008	0.032	0.813	0.016	0.031	0.031	0.165
paraphrase-multilingual-mpnet-base-v2	50	0.008	0.038	0.958	0.019	0.028	0.028	0.183
openai_embed_3_small	50	0.030	0.039	1.000	0.020	0.021	0.021	0.178
paraphrase-multilingual-MiniLM-L12-v2	50	0.007	0.038	0.958	0.019	0.031	0.031	0.187
multilingual-e5-large-instruct	50	0.009	0.039	1.000	0.020	0.021	0.021	0.179
KoSimCSE-roberta	50	0.008	0.038	0.958	0.019	0.032	0.032	0.188
openai	50	0.030	0.039	1.000	0.020	0.021	0.021	0.179
upstage_embed	50	0.076	0.039	1.000	0.020	0.020	0.020	0.177

**Table 5:** Results of quantitative analysis for sparse retriever: Korean Diabetes Association guideline.

Module_Name	Tokenizer	Top_k	Execution Time	F1-Score	Recall	Precision	MAP	MRR	NDCG
bm25	ko_kiwi	1	1.097	0.306	0.306	0.306	0.306	0.306	0.306
bm25	ko_kiwi	3	1.083	0.224	0.449	0.15	0.371	0.371	0.391
bm25	ko_kiwi	5	1.068	0.177	0.531	0.106	0.39	0.39	0.425
bm25	ko_kiwi	10	1.093	0.115	0.633	0.063	0.405	0.405	0.459
bm25	ko_kiwi	50	1.062	0.038	0.98	0.02	0.425	0.425	0.542
bm25	porter_stemmer	1	0.001	0.245	0.245	0.245	0.245	0.245	0.245
bm25	porter_stemmer	3	0.001	0.214	0.429	0.143	0.327	0.327	0.353
bm25	porter_stemmer	5	0.002	0.184	0.551	0.110	0.355	0.355	0.404
bm25	porter_stemmer	10	0.002	0.126	0.694	0.069	0.375	0.375	0.451
bm25	porter_stemmer	50	0.006	0.035	0.898	0.018	0.386	0.386	0.497

Mean average precision (MAP); mean reciprocal rank (MRR); and normalized discounted cumulative gain (NDCG).

**Table 6:** Results of quantitative analysis for sparse retriever: American Diabetes Association guideline.

Module_Name	Tokenizer	Top_k	Execution Time	f1-Score	Recall	Precision	MAP	MRR	NDCG
bm25	ko_kiwi	1	1.093	0.542	0.542	0.542	0.542	0.542	0.542
bm25	ko_kiwi	3	1.087	0.354	0.708	0.236	0.622	0.622	0.644
bm25	ko_kiwi	5	1.087	0.264	0.792	0.158	0.642	0.642	0.680
bm25	ko_kiwi	10	1.088	0.163	0.896	0.090	0.657	0.657	0.714
bm25	ko_kiwi	50	1.094	0.038	0.979	0.020	0.663	0.663	0.736
bm25	porter_stemmer	1	0.001	0.500	0.500	0.500	0.500	0.500	0.500
bm25	porter_stemmer	3	0.001	0.365	0.729	0.243	0.608	0.608	0.639
bm25	porter_stemmer	5	0.001	0.285	0.854	0.171	0.638	0.638	0.692
bm25	porter_stemmer	10	0.001	0.178	0.979	0.098	0.656	0.656	0.734
bm25	porter_stemmer	50	0.005	0.039	1.000	0.020	0.657	0.657	0.738

Mean average precision (MAP); mean reciprocal rank (MRR); and normalized discounted cumulative gain (NDCG).

## Data Availability

The Korean Diabetes Association’s 2023 Clinical Practice Guidelines are publicly available for download at https://www.diabetes.or.kr/bbs/?code=guide&mode=view&number=1284&page=1&code=guide (accessed on 26 September 2024). Additional data presented in this study are available upon reasonable request to the corresponding author.

## References

[1] Raffel C., Shazeer N., Roberts A., Lee K., Narang S., Matena M., Zhou Y., Li W., Liu P.J. (2020). Exploring the limits of transfer learning with a unified text-to-text transformer. J. Mach. Learn. Res..

[2] Touvron H., Lavril T., Izacard G., Martinet X., Lachaux M.-A., Lacroix T., Rozière B., Goyal N., Hambro E., Azhar F. (2023). Llama: Open and efficient foundation language models. arXiv.

[3] Achiam J., Adler S., Agarwal S., Ahmad L., Akkaya I., Aleman F.L., Almeida D., Altenschmidt J., Altman S., Anadkat S. (2023). Gpt-4 technical report. arXiv.

[4] Bai Y., Kadavath S., Kundu S., Askell A., Kernion J., Jones A., Chen A., Goldie A., Mirhoseini A., McKinnon C. (2022). Constitutional ai: Harmlessness from ai feedback. arXiv.

[5] Pal A., Umapathi L.K., Sankarasubbu M. (2023). Med-halt: Medical domain hallucination test for large language models. arXiv.

[6] Bender E.M., Gebru T., McMillan-Major A., Shmitchell S. On the dangers of stochastic parrots: Can language models be too big?. Proceedings of the 2021 ACM Conference on Fairness, Accountability, and Transparency.

[7] Perov V., Perova N. AI Hallucinations: Is “Artificial Evil” Possible?. Proceedings of the 2024 IEEE Ural-Siberian Conference on Biomedical Engineering, Radioelectronics and Information Technology (USBEREIT).

[8] Ji Z., Lee N., Frieske R., Yu T., Su D., Xu Y., Ishii E., Bang Y.J., Madotto A., Fung P. (2023). Survey of hallucination in natural language generation. ACM Comput. Surv..

[9] Kim C.Y., Kim S.Y., Cho S.H., Kim Y.-M. Bridging the language gap: Domain-specific dataset construction for medical LLMs. Proceedings of the International Joint Conference on Artificial Intelligence.

[10] Li X.L., Liang P. (2021). Prefix-tuning: Optimizing continuous prompts for generation. arXiv.

[11] Hu E.J., Shen Y., Wallis P., Allen-Zhu Z., Li Y., Wang S., Wang L., Chen W. (2021). Lora: Low-rank adaptation of large language models. arXiv.

[12] Hu Z., Wang L., Lan Y., Xu W., Lim E.-P., Bing L., Xu X., Poria S., Lee R.K.-W. (2023). Llm-adapters: An adapter family for parameter-efficient fine-tuning of large language models. arXiv.

[13] Xu X., Li M., Tao C., Shen T., Cheng R., Li J., Xu C., Tao D., Zhou T. (2024). A survey on knowledge distillation of large language models. arXiv.

[14] Zhang B., Liu Z., Cherry C., Firat O. (2024). When scaling meets llm finetuning: The effect of data, model and finetuning method. arXiv.

[15] Ding N., Qin Y., Yang G., Wei F., Yang Z., Su Y., Hu S., Chen Y., Chan C.-M., Chen W. (2023). Parameter-efficient fine-tuning of large-scale pre-trained language models. Nat. Mach. Intell..

[16] Moon J.S., Kang S., Choi J.H., Lee K.A., Moon J.H., Chon S., Kim D.J., Kim H.J., Seo J.A., Kim M.K. (2024). 2023 Clinical Practice Guidelines for Diabetes Management in Korea: Full Version Recommendation of the Korean Diabetes Association. Diabetes Metab. J..

[17] Care D. (2023). Standards of care in diabetes—2023. Diabetes Care.

[18] Ghanbari Haez S., Segala M., Bellan P., Magnolini S., Sanna L., Consolandi M., Dragoni M. A Retrieval-Augmented Generation Strategy to Enhance Medical Chatbot Reliability. Proceedings of the International Conference on Artificial Intelligence in Medicine.

[19] Karpukhin V., Oğuz B., Min S., Lewis P., Wu L., Edunov S., Chen D., Yih W.-T. (2020). Dense passage retrieval for open-domain question answering. arXiv.

[20] Ge J., Sun S., Owens J., Galvez V., Gologorskaya O., Lai J.C., Pletcher M.J., Lai K. (2024). Development of a liver disease-Specific large language model chat Interface using retrieval augmented generation. Hepatology.

[21] Matsumoto N., Moran J., Choi H., Hernandez M.E., Venkatesan M., Wang P., Moore J.H. (2024). KRAGEN: A knowledge graph-enhanced RAG framework for biomedical problem solving using large language models. Bioinformatics.

[22] Kim D., Kim B., Han D., Eibich M. (2024). AutoRAG: Automated Framework for optimization of Retrieval Augmented Generation Pipeline. arXiv.

